# Acalabrutinib in management of chronic lymphocytic leukemia: An Indian perspective

**DOI:** 10.1002/jha2.227

**Published:** 2021-05-28

**Authors:** Nitin Sood, Abraham Varghese, Joydeep Chakrabarty, Subhash Chezhian, Pranav Sopory

**Affiliations:** ^1^ Department of Medical Oncology and Haematology Medanta‐The Medicity Gurgaon Haryana India; ^2^ Department of Haemato‐Oncology and Clinical Haematology Little Flower Hospital and Research Centre Angamaly Aluva Kerala; ^3^ Department of Hematology and Bone Marrow Transplant HCG EKO Cancer Centre Kolkata India; ^4^ Department of Haematology Haemato‐oncology and Bone Marrow Transplant, MIOT Hospitals Chennai Tamil Nadu India; ^5^ Medical Affairs AstraZeneca Pharma India Ltd Bangalore India

## Abstract

The treatment landscape of chronic lymphocytic leukemia (CLL) has witnessed immense changes in the past decade. Several newer target therapies and their combinations with anti‐CD 20 therapies have got approval for management of CLL in the treatment‐naïve and relapsed/refractory setting. Also, the availability of newer diagnostic techniques has helped differentiate the disease into high‐ and low‐risk CLL which acts not just as a prognostic marker but also helps decide the best drug management that can be administered to the patients. Targeted therapy has largely overtaken chemoimmunotherapy in the management of CLL, except for a small subset of the population (young and fit with IGHV mutation). However, with targeted therapy, there is also an issue of previously uncommon treatment‐emergent adverse events, the duration of therapy, and financial toxicity. The aim of this review article is to gather results from all landmark CLL trials and discuss the feasibility of incorporating Acalabrutinib in the CLL landscape from an Indian perspective.

## 1 INTRODUCTION

The treatment of chronic lymphocytic leukemia (CLL) has undergone a transformative change in recent years. Treatment of newly diagnosed CLL has shifted from chemotherapy‐based regimens a decade ago to targeted therapies in the current era. Most treatment naïve CLL patients will receive either a Bruton tyrosine kinase (BTK) inhibitor or a B‐cell lymphoma 2 (BCL 2) inhibitor at some point in their treatment cycle. The treatment of CLL in resource‐constrained settings, where the majority of treatment cost comes from “out‐of‐pocket” expenditure, adds another dimension to the choice of treatment in a particular patient. This review focuses on the unique challenges posed by such cost constraints and looks at the evidence for the newly introduced Acalabrutinib to the mix of therapies. It also looks at the evidence for use of this drug both in frontline and relapse settings.

CLL predominantly affects older individuals, the median age of diagnosis is 72 years old [1]. CLL rates are higher in Western Europe and North America compared to Asia, where the incidence is generally low. The incidence of CLL in India is 0.41 per 100,000, approximately 10 times lower than the United States [2]. As per International Workshop on CLL (iwCLL) recommendations, diagnosing CLL requires the presence of ≥5 × 10^9^/L B‐lymphocytes in the peripheral blood, sustained for at least 3 months. The clonality of these B‐lymphocytes should be demonstrated by immunoglobulin light chain restriction using flow cytometry [3].

CLL is characterized by proliferation as well as accumulation of small, mature‐appearing monoclonal CD5^+^ B cells that express CD5, CD19, CD23, and CD20 (low), and exhibit immunoglobulin light‐chain restriction (kappa or lambda).

Patients with deletion of the short arm of chromosome 17, that is, del(17p), found in 5% to 8% of treatment naïve patients and in higher rates in relapsed/refractory patients, have shorter survival than patients with normal cytogenetics [4]. Del(17p) usually includes *TP53*, a prominent tumor suppressor gene, and is associated with poor response to chemotherapy‐immunotherapy regimens [5]. Patients without *del(17p)* but harboring a *TP53* mutation also have similar poor survival and deleterious outcome on chemotherapy. Patients with CLL cells that have a deletion in the long arm of chromosome 13, that is, del(13q14.3), which is the most common deletion found in CLL patients (approximately 55%) [6], have longer survival compared with patients that have normal karyotypes [7]. Patients with CLL cells that have *del[11q]*, a deletion of part of the long arm of chromosome 11, harboring the gene *ATM*, have shorter survival than patients with normal karyotypes. The absence of *ATM* gene makes the disease less susceptible to chemotherapy [8]. CLL cells expressing unmutated *IGHV*, that is, <98% cut‐off of *IGHV* identity to the germline counterpart are associated with more aggressive disease, with patients having shorter survival and poorer prognosis than those with mutated *IGHV*. This is extremely pronounced when treating patients with chemoimmunotherapy where M‐CLL patients without any cytogenetic poor risk factors have a significantly longer progression‐free survival (PFS) [9]. This probably is not true with newer agents like BTK inhibitors where *IGHV* status does not influence the efficacy [10].

## 2 TYPES OF TREATMENT MODALITIES

Table 1 summarizes the CLL treatment modalities accessible to Indian patients.

**TABLE 1:** CLL treatment modalities

Chemotherapies	‐ Purine analogues: Fludarabine, cladribine, pentostatin ‐ Alkylating agents: Bendamustine, chlorambucil, cyclophosphamide
Immunotherapies	‐ Anti‐CD20: Rituximab, obinutuzumab, ofatumumab ‐ Anti‐CD52: Alemtuzumab
Targeted therapy	‐ BTK inhibitors (ibrutinib, acalabrutinib) ‐ BCL‐2 inhibitors (venetoclax) ‐ PI3K inhibitors (idelalisib, duvelisib)
Combination regimens	‐ Fludarabine, cyclophosphamide, and rituximab ‐ Bendamustine and rituximab ‐ Acalabrutinib and obinutuzumab ‐ Venetoclax and obinutuzumab ‐ Venetoclax and rituximab ‐ Chlorambucil and obinutuzumab ‐ Chlorambucil and rituximab ‐ Ibrutinib and rituximab ‐ Ibrutinib and obinutuzumab ‐ Idelalisib and rituximab ‐ High‐dose methylprednisolone and rituximab ‐ Fludarabine, cyclophosphamide, and ofatumumab ‐ Lenalidomide^a^ and rituximab

Abbreviations: BTK, Bruton's tyrosine kinase; CD, cluster of differentiation; PI3K, phosphoinositide 3‐kinase.

^a^ Classified as an immunomodulatory agent.

## 3 FIRST‐LINE CLL TREATMENT PARADIGM

In the 2000s, chemoimmunotherapies (CITs) transformed the CLL treatment landscape, becoming the preferred approach for the first‐line treatment of CLL. However, individuals with *TP53*/del(17p) mutation do not respond well to chemotherapy‐containing regimens leaving an unmet need in this and other high‐risk populations (unmutated *IGHV*) defined in the chemotherapy era [11]. Therapies targeting PI3K, BTK, and BCL‐2, alone and/or in combination with anti‐CD20 antibodies, are driving innovations in the current landscape, providing increased efficacy and safety in the first‐line treatment of high‐risk patients. Results are shown in Table 2 (for chemotherapy) and Table 3 (for targeted therapy).

**TABLE 2:** Chemotherapy Regimens in treatment‐naïve CLL

Study	Overview	Key efficacy results
CLL8[12]	Phase 3 study of FCR vs FC	mPFS: 56.8 and 32.9 months for the FCR and FC group (HR, 0.59; 95% CI, 0.50‐0.69, *P* << .001) mOS: NR for FCR versus 86.0 months for the FC group (HR, 0.68; 95% CI, 0.54‐0.89, *P* = .001)
CLL10[13]	Phase 3 noninferiority trial of first‐line BR vs FCR in patients without del(17p)	mPFS: 41·7 months (95% CI 34·9‐45·3) with BR and 55·2 months (95% CI not evaluable) with FCR (HR 1·643, 90·4% CI 1·308‐2·064). BR did not pass noninferiority analysis compared by FCR, but BR was associated with fewer toxic effects.

Abbreviations: BR, bendamustine + rituximab; CR, complete remission; FC, fludarabine, cyclophosphamide; FCR, fludarabine, cyclophosphamide, and rituximab; HR, hazard ratio; mOS, median overall survival; mPFS, median progression‐free survival; MRD, minimal residual disease; NR, not reached; OS, overall survival; PFS: Progression‐free survival

**TABLE 3:** Targeted therapy regimens in treatment‐naïve CLL

Study	Overview	Key efficacy results
RESONATE‐2[14]	Phase 3 study of ibrutinib vs chlorambucil	mPFS: NR and 15.0 months for ibrutinib and chlorambucil group (HR, 0.146; 95% CI, 0.098‐0.218; *P* < .0001) 5‐year PFS rate: 70% and 12% for ibrutinib and chlorambucil (HR, 0.146; 95% CI, 0.098‐0.218; *P* < .0001) 5‐year OS rate: 83% and 68% for ibrutinib and chlorambucil (HR, 0.450; 95% CI, 0.266–0.761)
ECOG 1912[15]	Phase 3 study of ibrutinib plus rituximab vs FCR	3‐year PFS rate: 89.4% and 72.9% for ibrutinib plus rituximab and FCR (HR, 0.35; 95% CI, 0.22 to 0.56; *P* < .001) 3‐year OS rate: 98.8% and 91.5% for ibrutinib plus rituximab and FCR at 3 years; (HR, 0.17; 95% CI, 0.05 to 0.54; *P* < .001)
ALLIANCE[16]	Phase 3 study of BR vs ibrutinib vs ibrutinib plus rituximab	2‐year PFS rate: 74%, 87% and 88% for BR, ibrutinib* alone and ibrutinib plus rituximab**; *(HR, 0.39; 95% CI, 0.26 to 0.58; *P* < .001); **(HR, 0.38; 95% CI, 0.25 to 0.59; *P* < .001) mPFS: 43 months for BR and NR for ibrutinib and ibrutinib plus rituximab. There was no significant difference between the ibrutinib‐plus‐rituximab and the ibrutinib group with regard to progression‐free survival (HR, 1.00; 95% CI, 0.62 to 1.62; p = 0.49)
CLL14[17]	Phase 3 study of venetoclax plus obinutuzumab vs chlorambucil plus obinutuzumab	2‐year PFS rate: 88.2% and 64.1% for venetoclax plus obinutuzumab and chlorambucil plus obinutuzumab (HR, 0.35; 95% CI, 0.23 to 0.53; *P* < .001). 2‐year OS rate : 91.8% and 93.3% for venetoclax plus obinutuzumab and chlorambucil plus obinutuzumab (HR = 1.24; 95% CI, 0.64 to 2.40; p = 0.52)
iLLUMINATE[18]	Phase 3 study of ibrutinib plus obinutuzumab vs chlorambucil plus obinutuzumab	mPFS: NR and 19.0 months for ibrutinib plus obinutuzumab and chlorambucil plus obinutuzumab (HR, 0.23; 95% CI, 0.15 to 0.37; *P* < .0001)

## 4 RELAPSED/REFRACTORY CLL TREATMENT PARADIGM

Relapsed/refractory CLL is more aggressive than the treatment‐naïve counterpart and more resistant to chemoimmunotherapy partly because of higher rates of del17p and other mutations due to clonal evolution [19]. It is therefore necessary for patients to be re‐tested and check if the mutation profile has changed. In the past decade, management of relapsed/refractory CLL has shifted to BTK inhibitors and BCL‐2 inhibitors. Table 4 shows efficacy results for the two clinical trials in relapsed/refractory setting.

**TABLE 4:** Treatment paradigm for Relapsed/Refractory CLL

Study	Overview	Key efficacy results
MURANO[20, 21]	Phase 3 study of venetoclax plus rituximab vs BR	2‐year PFS rate: 84.9% and 36.3% for venetoclax plus rituximab and BR (HR, 0.17; 95% CI, 0.11 to 0.25; *P* < .001) by the stratified log‐rank test). 2‐year OS rate: 91.9% and 86.6% for venetoclax plus rituximab and BR (HR, 0.48; 95% CI, 0.25 to 0.90)
RESONATE[22]	Phase 3 study of ibrutinib vs ofatumumab	mPFS: 44.1 vs 8.1 months (HR, 0.148; 95% CI, 0.113‐0.196; *P *˂ .001) median OS: 67.7 months and 65.1 months for ibrutinib ofatumumab, irrespective of the extensive (68%) crossover to ibrutinib (HR, 0.810; 95% CI, 0.602‐1.091)

Abbreviations: FCR, fludarabine, cyclophosphamide, and rituximab; HR, hazard ratio; mOS, median overall survival; mPFS, median progression‐free survival; MRD, minimal residual disease; NR, not reached; OS, overall survival; PFS: Progression‐free survival.

## 5 ACALABRUTINIB

**FIGURE 1:**
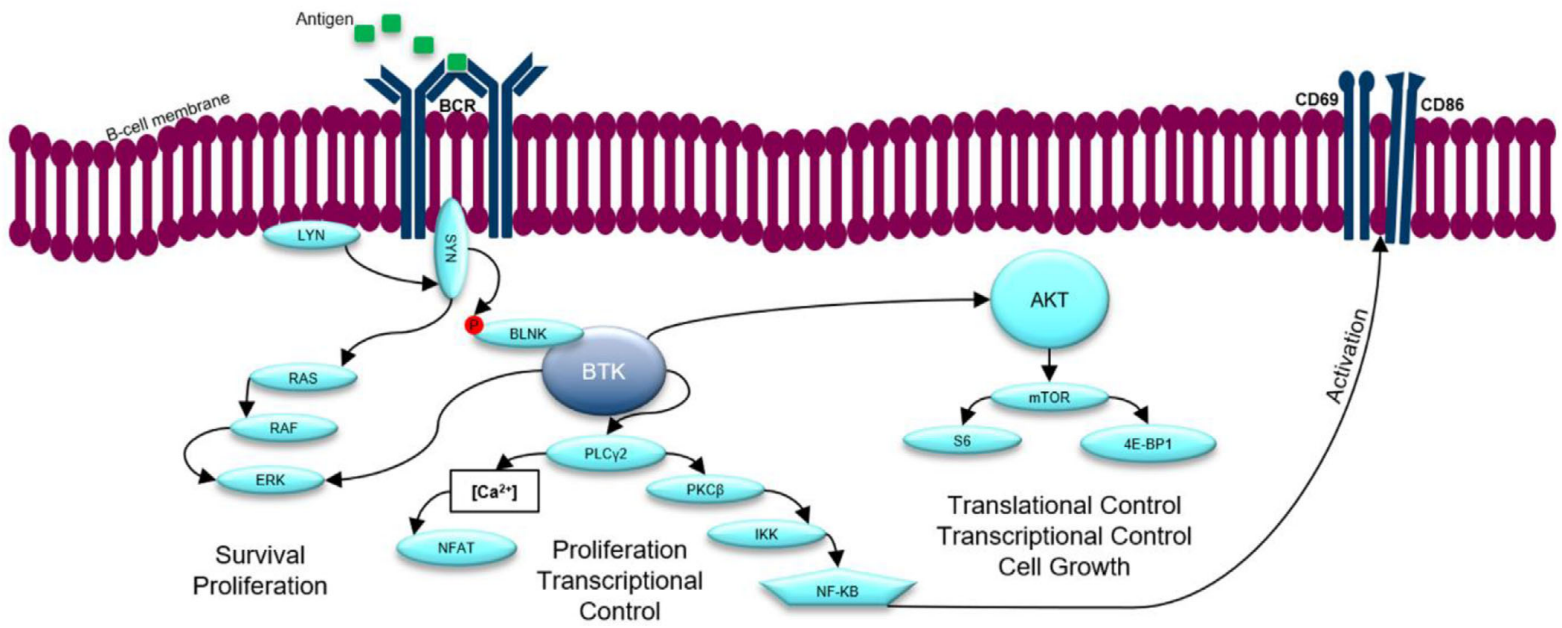
BTK plays a key role in the B cell receptor, or BCR, pathway which regulates the development, function, and survival of the B cell. BTK has been implicated in the pathogenesis of B cell malignancies—including chronic lymphocytic leukemia, or CLL—and has recently emerged as an important therapeutic target. In a malignant or cancerous B cell, BTK may become overexpressed and persistently activated, allowing the cell to thrive in the peripheral blood, bone marrow, and lymph nodes. BCR, B‐cell receptor; BLNK, B‐cell linker; BTK, Bruton tyrosine kinase; CD, cluster of differentiation; IKK, I kappa B kinase; LCg2, phospholipase Cg2; LYN, Lck/Yes novel tyrosine kinase; mTOR, mammalian target of rapamycin; NF‐kB, nuclear factor kappa‐light chain enhancer of activated B‐cells; NFAT, nuclear factor of activated T‐cells; PKC, protein kinase C; PLC, phospholipase C; SYK, spleen tyrosine kinase

BTK is a non‐receptor protein tyrosine kinase that plays a critical role in B‐cell development, differentiation, signaling, proliferation, and survival. It is a key component of the BCR signaling pathway and also functions in the TLR, G protein‐coupled chemokine receptor, B‐cell adhesion, and migration signaling pathways (Figure 1) [23].

Acalabrutinib and its active metabolite, ACP‐ 5862, inhibit BTK enzymatic activity irreversibly by covalently binding a cysteine residue in the BT active site. This inhibits BTK‐mediated activation of downstream signaling proteins CD86 and CD 69 as well as malignant B‐cell proliferation and survival. Acalabrutinib has been shown to be more selective than ibrutinib and is hence considered a safer option for patients requiring treatment in CLL [24]. In an in vivo IC_50_ kinase inhibition study [25], the affinity for BTK was highest among the enzymes tested in the panel (Table 5). Acalabrutinib IC_50_ concentrations for ITK (tyrosine‐protein kinase) and EGFR (epidermal growth factor receptor) were more than 200‐fold higher (less inhibition) than for BTK. Concentrations for ERBB4 (Erb‐B2 Receptor Tyrosine Kinase 4) and BMX (bone marrow tyrosine kinase gene in chromosome X) were three‐ and ninefold higher (less inhibition) than for BTK.

**TABLE 5:** Potency of acalabrutinib measured in an IC_50_ inhibition kinase study

Kinase	Acalabrutinib IC_50_ concentration
BTK	5.1
ITK	>1000
TEC	126
BMX	46
TXK	368
BLK	>1000
ERBB2	∼1000
ERBB4	16
EGFR	>1000
JAK3	>1000

Abbreviations: BTK, Bruton tyrosine kinase; ITK, tyrosine‐protein kinase; TEK, tyrosine kinase expressed in hepatocellular carcinoma; BMX, bone marrow tyrosine kinase gene in chromosome X; TXK, T and X cell expressed kinase; BLK, B‐lymphocyte kinase; ERBB, erythroblastosis oncogene B; EGFR, epidermal growth factor receptor; JAK, janus‐associated kinase.

Safety and efficacy of acalabrutinib were revealed in two randomized, open‐label, multi‐center phase III trials; the ELEVATE‐TN trial in treatment‐naïve and ASCEND trial in relapsed/refractory CLL. In the ELEVATE‐TN trial, patients who received acalabrutinib in combination with obinutuzumab or acalabrutinib monotherapy had improved efficacy parameters compared to the patients who received obinutuzumab in combination with chlorambucil [26]. After a median follow‐up of 28 months, acalabrutinib‐obinutuzumab combination arm had a 90% reduction in relative risk of progression or death with had a significantly longer mPFS (NR, 95% CI NE‐NE) compared the obinutuzumab‐chlorambucil arm (22.6 months, 20.2‐27.6), (HR: 0·10, 0·06–0·17; *P* < .0001). Acalabrutinib monotherapy (secondary endpoint) was also associated with statistically improved PFS over obinutuzumab‐chlorambucil (HR 0·20, 95% CI 0·13–0·30; *P* < .0001). There was consistent PFS benefit with acalabrutinib dual therapy and monotherapy in the prespecified (including high‐risk) subgroups over obinutuzumab‐chlorambucil. The frequency of discontinuations due to adverse events was not higher in the acalabrutinib‐containing groups despite the longer treatment durations (11% in the acalabrutinib‐obinutuzumab group, 9% in the acalabrutinib monotherapy group, and 14% in the obinutuzumab‐chlorambucil group). In the ASCEND trial for relapsed/refractory CLL, acalabrutinib monotherapy showed improved efficacy over investigator's choice of therapy in combination with rituximab; idelalisib (I‐R) or bendamustine (B‐R) [27]. mPFS was NR in the acalabrutinib monotherapy arm versus 16.5 months in the comparator arm (95% CI, 14.0 to 17.1 months). The relative reduction in the risk of progression or death was 69% with acalabrutinib monotherapy (HR, 0.31; 95% CI, 0.20 to 0.49; *P* < .0001). Investigator assessed PFS in prespecified subgroups (including high‐risk) was improved with acalabrutinib monotherapy. Even though the median duration of exposure was longest in the acalabrutinib arm, adverse events (including serious adverse events were reported more frequently in the I‐R treatment arm than acalabrutinib monotherapy or B‐R treatment arm.

A retrospective analysis (January 2013 to October 2015) of 447 adult patients on CLL on ibrutinib revealed high rates of drug discontinuation [28]. At a median follow‐up of 20 months, roughly 50% of the study population had discontinued ibrutinib. Twenty‐three percent discontinued ibrutinib due to toxicity, the remaining discontinuing due to disease progression, death, or other reasons.

In a phase II study, 60 ibrutinib‐intolerant patients were administered acalabrutinib till disease progression or unacceptable toxicity [29]. The median duration of prior ibrutinib therapy was 6 months (range, <1 to 56). Common adverse events (>2 patients) that led to discontinuation of prior ibrutinib therapy were atrial fibrillation/flutter (28%), diarrhea (12%), rash (12%), and arthralgia (10%). At a median follow‐up of 23 months, there were still 48 patients on study. Although all patients discontinued prior ibrutinib due to adverse events, only 12% discontinued acalabrutinib due to adverse events after 23 months of follow‐up. An overall response rate of 72% (5% CR rate) was observed in this population. Acalabrutinib, therefore, is a viable option for patients who discontinue ibrutinib due to its toxicity. In a phase I/II study of acalabrutinib in relapsed/refractory CLL, at 42 months of follow‐up, adverse events of all grades occurring in ≥10% of patients receiving acalabrutinib; generally, these were diarrhea (52%), headache (51%), upper respiratory tract infection (37%), and fatigue (34%). The adverse events were mostly mild to moderate in severity and mostly self‐limiting [30].

## 6 INDIAN PERSPECTIVE

From the above data, it is evident that the majority of CLL patients benefit more from targeted treatment than chemoimmunotherapy (except in a small proportion of patients with *IGHV* mutation) both in terms of efficacy and toxicity. It is not unusual to experience considerable delays in the release of several of the new drugs to the developing world after it is licensed and widely available in the western world. There are several reasons for this delay; such as poor market performance due to the price, fear of being duplicated during the patency period, and fear of arbitrage if there is any cost reduction. One of the aims of this manuscript is to throw some light on the question of how acalabrutinib would fit into an LMIC (lower and middle income countries) context taking India as an example. Most LMIC healthcare systems are driven predominantly by out of pocket payments by patients, although insurance and various government schemes are increasingly playing significant roles. Newer drugs with their huge cost put additional pressure on most health systems particularly on the patient's pocket in the context of LMIC, but this cost has to be understood in the larger scheme. Chemotherapy agents, although cheaper on the drug cost, are associated with additional supportive care burden (febrile neutropenia, admission cost, administration charges, etc). For example, in India, the average cost of chemoimmunotherapy, such as FCR (fludarabine, cyclophosphamide, and rituximab) and BR (bendamustine and rituximab) is around US$ 900 per cycle and US$ 9000 for six cycles. For delivering a chemotherapy regimen like FCR, supportive care cost is high as the average cost of one febrile neutropenia admission is around US$ 2000. Since these costs are significant they may also tilt the cost‐benefit ratio toward the targeted agents especially if generic versions are available. Apart from the issue of cost, there is also a familiarity associated with chemoimmunotherapy agents as many hematologists have been using these combinations for several years. Some physicians claim that even though they are aware of the high risks associated with it, they are trained to manage the adverse events accordingly. These practicing habits are surely changing with time. At present, India is facing the second surge of COVID cases with 350 000‐500 000 cases being reported daily. In such challenging times, for a country like India where the healthcare sector is facing acute shortage of beds and ICU, it is important that the patients suffering from CLL maintain social distancing while staying safe and continue their fight against CLL via oral and safer targeted therapies. Second‐generation BTK inhibitors have a specific role in patients where Ibrutinib is not tolerated or has relative contraindication beyond the conventional indication for BTK inhibitors. BTK inhibitors are the preferred treatment in 17p deleted and or *TP53* mutated CLL and possibly in *IGHV* unmutated CLL. Cardiac toxicity is a major problem with first‐generation BTK inhibitor. In LMIC, there is an increasing trend of developing life‐style diseases and in fact cardiovascular diseases are even now more numerous in India and China than in all the economically developed countries in the world put together. Therefore, second‐generation BTK inhibitors may have a bigger role to play in these countries.

## 7 CONCLUSION

In this review article, we have discussed in detail the pathophysiology of CLL, the clinical staging systems, the key studies that have helped shape the treatment paradigm of CLL in the treatment‐naïve and relapsed/refractory setting. For more than half a century, chemotherapy was the treatment of choice for all CLL patients with an active disease. With the introduction of newer agents, the use of chemotherapy has declined significantly, except in the small proportion of young and fit patients with low‐risk CLL (*IGHV* mutated) where FCR regimen has shown remissions of 10 years or more. Newer and more selective agents have drastically improved the quality of life in patients with CLL, especially those with high‐risk CLL. Even though the BTK inhibitors (ibrutinib and acalabrutinib) generally do not induce a deep remission early on, significant survival advantage especially in terms of progression‐free has been noted when compared to the standard of treatment when they are continued till disease progression or development of treatment‐related adverse events. Although there have been no head‐to‐head trials, acalabrutinib, being a more selective inhibitor of BTK is considered safer and might lead to lesser adverse events and fewer discontinuations over the long term. In the two landmark trials of Acalabrutinib in the first‐line and relapsed/refractory setting (ELEVATE‐TN and ASCEND), it was observed to be more efficacious and safer than comparators as a monotherapy or in combination with an anti‐CD 20 inhibitor. Finite therapy has been established by the addition of venetoclax in combination with anti‐CD 20 antibody. However, more mature data is needed to know whether finite treatment with BCL‐2 inhibitor or indefinite treatment with BTK inhibitors is advantageous in terms of long‐term survival. Finally, we discussed how to implement acalabrutinib in an Indian setting.

## CONFLICT OF INTEREST

Pranav Sopory is an employee of AstraZeneca Pharma India Ltd.
